# Supplementary material for: Endothelial cell responses in sepsis are attenuated by targeting truncated procalcitonin
Source: Nat Commun. 2026 Jan 21;17:827. doi: 10.1038/s41467-025-68199-x (PMC12824222; doi:10.1038/s41467-025-68199-x)
Supplement: Supplementary file 1 — Supplementary Information [file 41467_2025_68199_MOESM1_ESM.pdf]

Supplementary Figure 1.

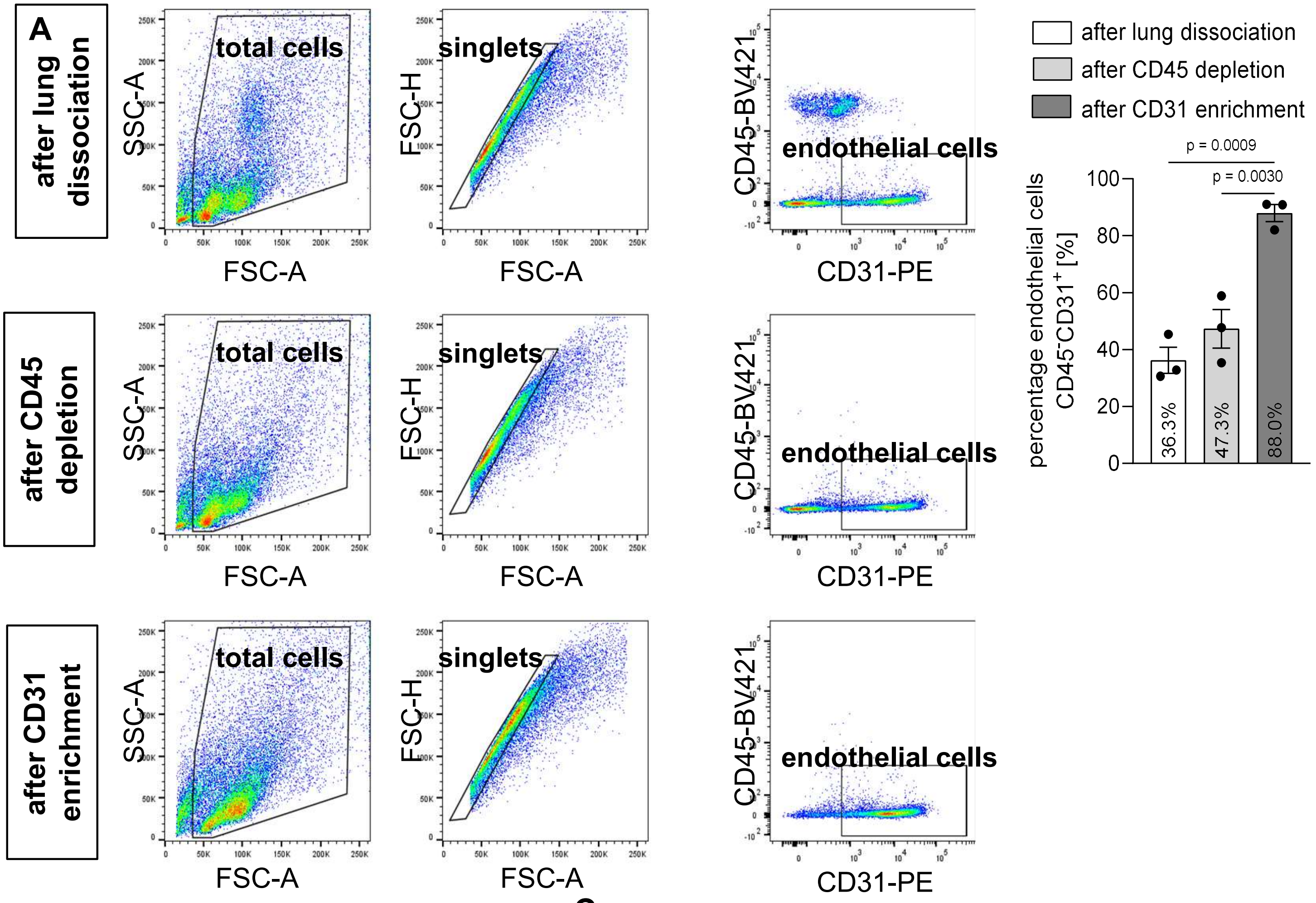

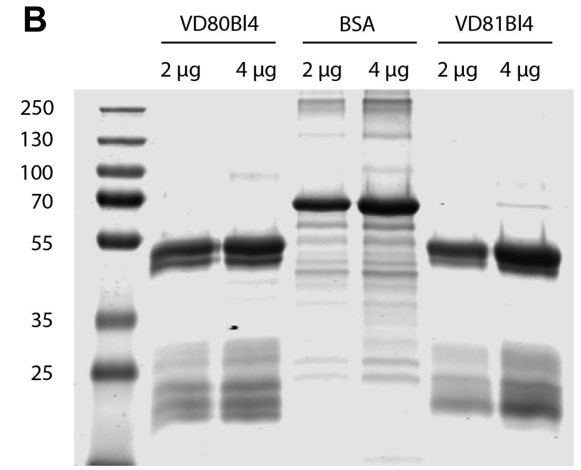

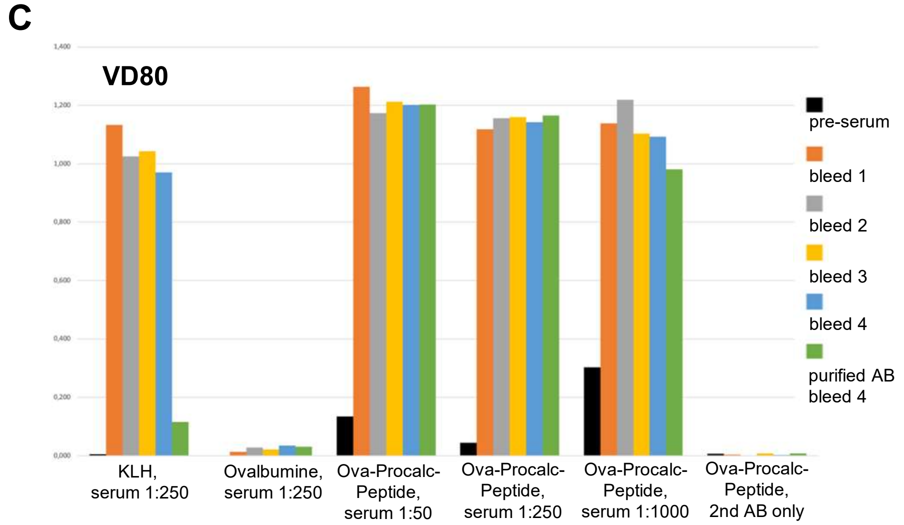

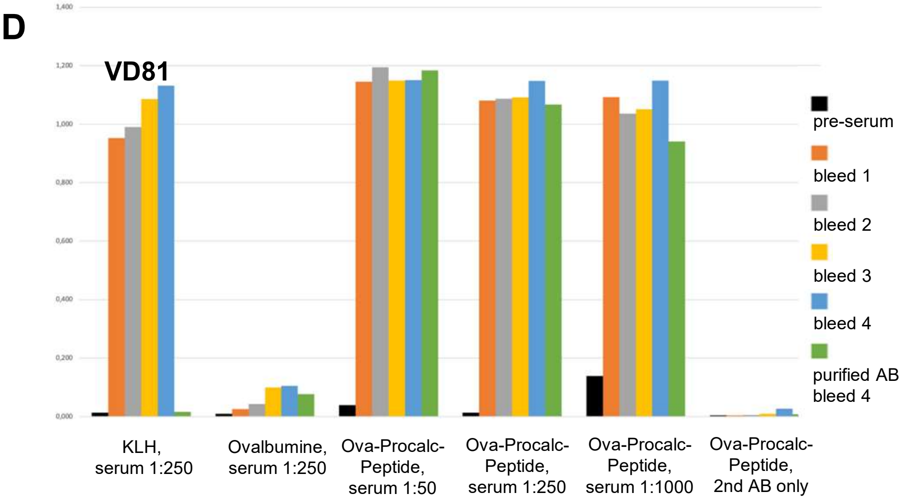

Supplementary Figure 1. A Purity of endothelial cells in differnt stages of sorting n=3, ordinary one way ANOVA/Bonferroni. Data presented as mean±SEM. **B** Uncropped Coomassie gel of purified antibodies from two rabbits (VD80Bl4 and VD81Bl4). **C**, **D** Results of affinity evaluation of sera and purified antibodies on surfaces coated with the immunogen (procalc-peptide) or controls. Source data are provided as a Source Data file.

Supplementary Figure 2.

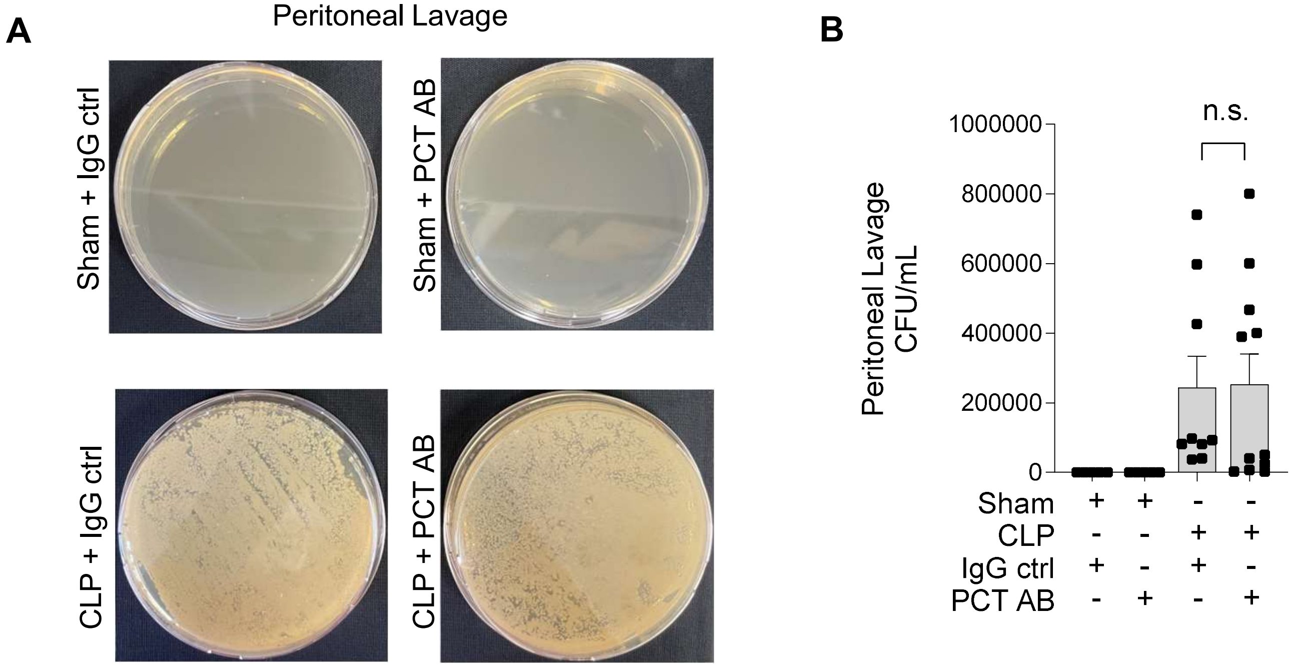

Supplementary Figure 2. A Representative photographs showing agar plates of murine blood after incubation at 37°C for 24hours. **B** Colony forming unit count in peritoneal lavange, culture withdrawn 18hours after sepsis induction by cecal ligation and puncture (CLP)/control (Sham) following injection of the antibody or respective control IgG, n=5 (Sham+IgG, Sham+AB), n=9 (CLP+IgG), n=11(CLP+AB) mice/group. One-way ANOVAA/Bonferroni. Data presented as mean±SEM Source data are provided as a Source Data file.

Supplementary Figure 3.

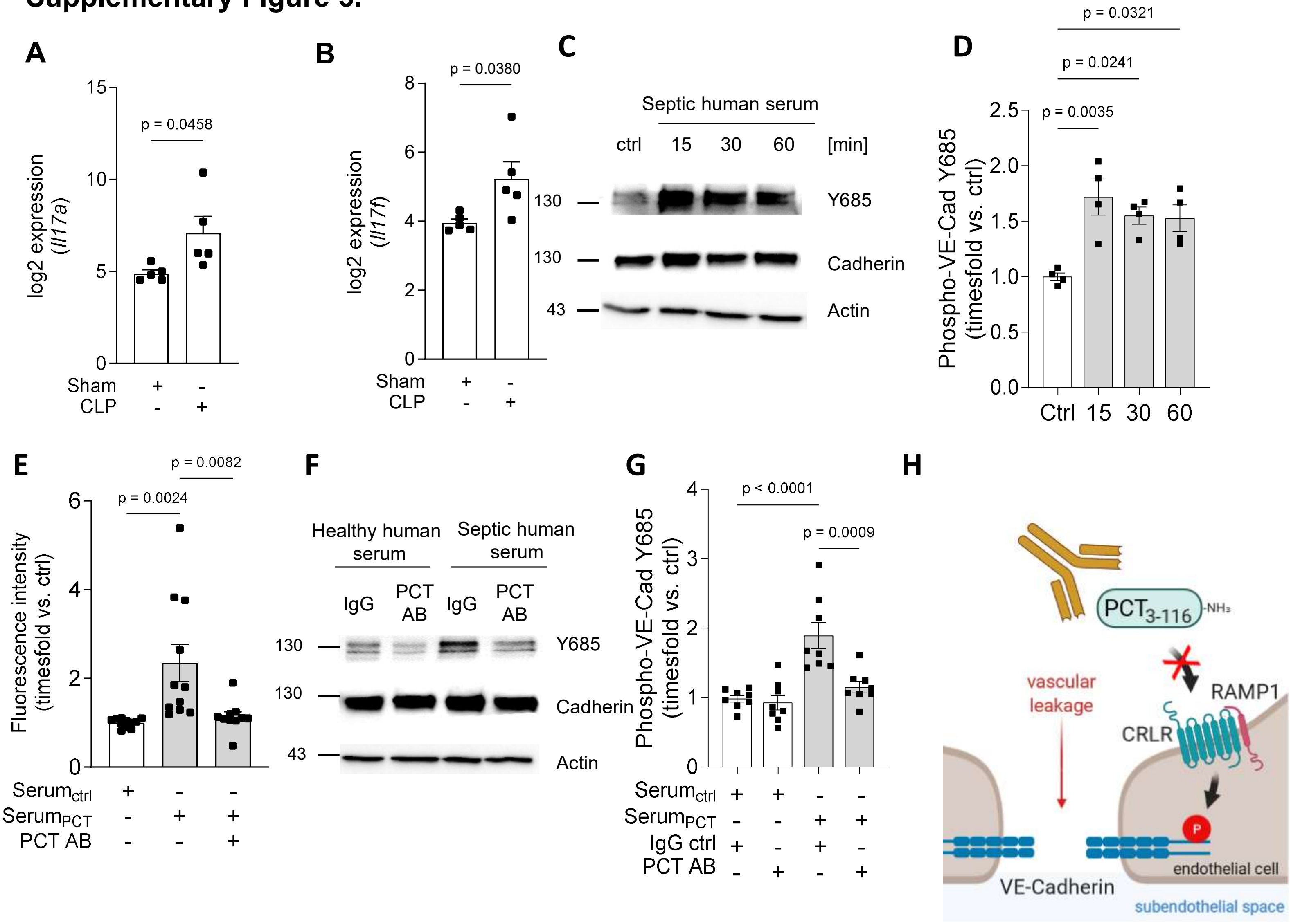

Supplementary Figure 3. A, **B** Expression of genes of the interleukin-17 (*Il17*) family in endothelial cells from septic mice, n=5 One-way ANOVA/Bonferroni. **C,D** VE-Cadherin phosphorylation is increased in human endothelial cells upon stimulation with septic serum of one patient (n=4 individual technical replicates). One-way-ANOVA/Bonferroni. **E** Increased endothelial cell permeability after septic human serum stimulation is reduced by an antibody targeting procalcitonins N-terminus that resulted in a reduction of VE-Cadherin phosphorylation in response to septic patients plasma (E: n=11 $Serum_{ctrl}$ and $Serum_{PCT}$, n=10 $Serum_{PCT}$+AB individual technical replicates; **F, G**, n=8 individual technical replicates). **H** Schematic overview. Created in BioRender. Brabenec, L. (2025) https://BioRender.com/odd3os6. Data presented as mean±SEM. Source data are provided as a Source Data file.
