# Supplementary material for: Endothelial cell responses in sepsis are attenuated by targeting truncated procalcitonin
Source: Nat Commun. 2026 Jan 21;17:827. doi: 10.1038/s41467-025-68199-x (PMC12824222; doi:10.1038/s41467-025-68199-x)
Supplement: Supplementary file 2 — Reporting Summary [file 41467_2025_68199_MOESM2_ESM.pdf]

Corresponding author(s): Nana-Maria Wagner
Last updated by author(s): Nov 9, 2025

Reporting Summary

Nature Portfolio wishes to improve the reproducibility of the work that we publish. This form provides structure for consistency and transparency in reporting. For further information on Nature Portfolio policies, see our Editorial Policies and the Editorial Policy Checklist.

Statistics

For all statistical analyses, confirm that the following items are present in the figure legend, table legend, main text, or Methods section.

n/a	Confirmed	
☐	☒	The exact sample size (*n*) for each experimental group/condition, given as a discrete number and unit of measurement
☐	☒	A statement on whether measurements were taken from distinct samples or whether the same sample was measured repeatedly
☐	☒	The statistical test(s) used AND whether they are one- or two-sided<br>*Only common tests should be described solely by name; describe more complex techniques in the Methods section.*
☒	☐	A description of all covariates tested
☐	☒	A description of any assumptions or corrections, such as tests of normality and adjustment for multiple comparisons
☐	☒	A full description of the statistical parameters including central tendency (e.g. means) or other basic estimates (e.g. regression coefficient) AND variation (e.g. standard deviation) or associated estimates of uncertainty (e.g. confidence intervals)
☐	☒	For null hypothesis testing, the test statistic (e.g. F, t, r) with confidence intervals, effect sizes, degrees of freedom and P value noted<br>*Give P values as exact values whenever suitable.*
☒	☐	For Bayesian analysis, information on the choice of priors and Markov chain Monte Carlo settings
☒	☐	For hierarchical and complex designs, identification of the appropriate level for tests and full reporting of outcomes
☒	☐	Estimates of effect sizes (e.g. Cohen's *d*, Pearson's *r*), indicating how they were calculated

Our web collection on statistics for biologists contains articles on many of the points above.

Software and code

Policy information about availability of computer code

Data collection	Trimgalore Version0.4.4, FastQC version 0.11.4, FACS Diva
Data analysis	Graphpad Prism Version 10.6.1, Graphpad Prism Version 7, Image J Win6, SPSS Statistics 14, FastQC (version 0.11.4)STAR (version 2.5.2b), R software package (version 4.2.1), pheatmap (version 1.0.12.), RsubRead (version 1.32.4) DESeq2 package (version 1.16.1)limma package (version 3.58.1),

For manuscripts utilizing custom algorithms or software that are central to the research but not yet described in published literature, software must be made available to editors and reviewers. We strongly encourage code deposition in a community repository (e.g. GitHub). See the Nature Portfolio guidelines for submitting code & software for further information.

Data

Policy information about availability of data

All manuscripts must include a data availability statement. This statement should provide the following information, where applicable:

- Accession codes, unique identifiers, or web links for publicly available datasets
- A description of any restrictions on data availability
- For clinical datasets or third party data, please ensure that the statement adheres to our policy

The data that support the findings of this study are mostly shown in the figures (single data points). Source data are provided with this paper. RNA sequencing data

that support the findings of this study have been deposited in GEO with the accession codes GSE244815 and GSE244943 (https://www.ncbi.nlm.nih.gov/geo/query/acc.cgi?acc=GSE244815, https://www.ncbi.nlm.nih.gov/ geo/query/acc.cgi?acc= GSE244943")

Research involving human participants, their data, or biological material

Policy information about studies with human participants or human data. See also policy information about sex, gender (identity/presentation), and sexual orientation and race, ethnicity and racism.

Reporting on sex and gender	No sex differences
Reporting on race, ethnicity, or other socially relevant groupings	N/A
Population characteristics	See above
Recruitment	Between February and November 2022, we recruited 19 adult patients from the intensive care units of the University Hostpital Munster who met the Sepsis-3 definition criteria and exhibited hyperprocalcitoninemia (plasma concentration >0.5 ng/mL) Written informed consent was obtained from patients or their legal representatives. The study received approval from the Ethics Committee of the University Hospital Munster (ID:2019-494-f-S) and was registered on ClinicalTrials.gov (registration number:NCT05703802, principal investigator: S. Kintrup, MD).<br>Patients were excluded, if they had immunological disorders, were taking immunosuppressive agents other than hydrocortisone, had viral or fungal sepsis, or had a terminal preexisting disease. Upon recruitment, 15 mL of serum were collected from each patient using two serum gel monovettes.
Ethics oversight	The study received approval from the Ethics Committee of the University Hospital Munster (ID:2019-494-f-S) and was registered on ClinicalTrials.gov (registration number:NCT05703802, principal investigator: S. Kintrup, MD).

Note that full information on the approval of the study protocol must also be provided in the manuscript.

Field-specific reporting

Please select the one below that is the best fit for your research. If you are not sure, read the appropriate sections before making your selection.

☒ Life sciences ☐ Behavioural & social sciences ☐ Ecological, evolutionary & environmental sciences

For a reference copy of the document with all sections, see nature.com/documents/nr-reporting-summary-flat.pdf

Life sciences study design

All studies must disclose on these points even when the disclosure is negative.

Sample size	Sample sizes were determined with Power analysis
Data exclusions	No data was excluded
Replication	confirmed, experiments were independently performed at least 3 times. If necessary experiments were perfored more often.
Randomization	all studies were randomized
Blinding	all studies were blinded

Reporting for specific materials, systems and methods

We require information from authors about some types of materials, experimental systems and methods used in many studies. Here, indicate whether each material, system or method listed is relevant to your study. If you are not sure if a list item applies to your research, read the appropriate section before selecting a response.

Materials & experimental systems

n/a	Involved in the study	
☐	☒	Antibodies
☒	☐	Eukaryotic cell lines
☒	☐	Palaeontology and archaeology
☐	☒	Animals and other organisms
☐	☒	Clinical data
☒	☐	Dual use research of concern
☒	☐	Plants

Methods

n/a	Involved in the study	
☒	☐	ChIP-seq
☐	☒	Flow cytometry
☒	☐	MRI-based neuroimaging

Antibodies

Antibodies used

VE-Cadherin at Tyrosine 685 (Abcam, ab119785) Polyclonal,Clone not specified, Dilution 1:1000,
total VE-Cadherin (Santa Cruz Biotech, sc9989) Product Clone Name: F-8, Dilution 1:200
b-actin (Thermofisher, MA5-15739) Clone: (BA3R), Dilution 1:10000
anti-rabbit IgG (7074S, Cell Signaling) Clone: not specified, Dilution 1:1000
anti-mouse IgG (7076S, Cell Signaling) Clone: not specifiedDilution 1:5000

Validation

Affinity purification Immunogen, according to manufacturers websites

Animals and other research organisms

Policy information about studies involving animals; ARRIVE guidelines recommended for reporting animal research, and Sex and Gender in Research

Laboratory animals

Wildtype mice C57BL/6J #000664, The Jackson Laboratory

Wild animals

N/A

Reporting on sex

No sex differences applicable. Experimental groups contain males and females

Field-collected samples

N/A

Ethics oversight

Animal experiments were approved by the governmental ethical board at the Animal Care and Use Committee of North Rhine Westphalia, germany, 81-02.04.2021.A396

Note that full information on the approval of the study protocol must also be provided in the manuscript.

Clinical data

Policy information about clinical studies
All manuscripts should comply with the ICMJE guidelines for publication of clinical research and a completed CONSORT checklist must be included with all submissions.

Clinical trial registration

Trial registered on ClinicalTrials.gov NCT05703802

Study protocol

https://clinicaltrials.gov/study/NCT05703802

Data collection

Between February and November 2022, we recruited 19 adult patients from the intensive care units of the University Hostpital Munster who met the Sepsis-3 definition criteria and exhibited hyperprocalcitoninemia (plasma concentration >0.5 ng/mL) Written informed consent was obtained from patients or their legal representatives. The study received approval from the Ethics Committee of the University Hospital Munster (ID:2019-494-f-S) and was registered on ClinicalTrials.gov (registration number:NCT05703802, principal investigator: S. Kintrup, MD).
Patients were excluded, if they had immunological disorders, were taking immunosuppressive agents other than hydrocortisone, had viral or fungal sepsis, or had a terminal preexisting disease. Upon recruitment, 15 mL of serum were collected from each patient using two serum gel monovettes

Outcomes

procalcitonin serum levels tested by Procalcitonin-variants ELISA-Assay

Plants

Seed stocks	N/A
Novel plant genotypes	N/A
Authentication	N/A

Flow Cytometry

Plots

Confirm that:

☒ The axis labels state the marker and fluorochrome used (e.g. CD4-FITC).

☒ The axis scales are clearly visible. Include numbers along axes only for bottom left plot of group (a 'group' is an analysis of identical markers).

☒ All plots are contour plots with outliers or pseudocolor plots.

☒ A numerical value for number of cells or percentage (with statistics) is provided.

Methodology

Sample preparation	murine lungs were excised 18h after sepsis induction. Dissociation of lung tissue into single cell suspensions for subsequent cell separations was performed using the miltenyi lung dissociation kit and standard protocol (130-095-927, Miltenyi biotec). Lungs were dissected and collected in a c-tube containing Enyyme A and D and Buffer S. The preinstalled lung dissociation protocol 37_m_LDK_1 on the miltenyo Octo Dissociator with heaters was used immediately for obtaining single cell culture. Endothelial cells were isolated from suspensions using MACS techology (Miltenyi biotec). Cells were resuspended in PEB (Phospate bufferd saline, 2mM EDTA, 0.5% Bovine Serum Albumine) buffer following depletion of CD45+ cells by using CD45-coupled microbeads. After 15 min. of incubation, cells were placed on a LS column in a magnetic separator and rinsed with PEB buffer three times. The CD45- fraction was collected and CD31 antibody -coupled beads were used for magnetic sorting of endothelial cells as before to obtain pure cell culture.
Instrument	BD Acurri 6
Software	FACS Diva
Cell population abundance	Purity was determined by step-by-step flow analysis
Gating strategy	Gating strategy is stated in the supplementary files

☒ Tick this box to confirm that a figure exemplifying the gating strategy is provided in the Supplementary Information.
